# Drug Resistance in *Salmonella enterica* ser. Typhimurium Bloodstream Infection, Malawi

**DOI:** 10.3201/eid2011.141175

**Published:** 2014-11

**Authors:** Nicholas A. Feasey, Amy K. Cain, Chisomo L. Msefula, Derek Pickard, Maaike Alaerts, Martin Aslett, Dean B. Everett, Theresa J. Allain, Gordon Dougan, Melita A. Gordon, Robert S. Heyderman, Robert A. Kingsley

**Affiliations:** Liverpool School of Tropical Medicine, Liverpool, UK (N.A. Feasey, R.S. Heyderman);; Wellcome Trust Sanger Institute, Cambridge, UK (N.A. Feasey, A.K Cain, D. Pickard, M. Aslett, G. Dougan, R.A. Kingsley);; University of Malawi College of Medicine, Blantyre, Malawi (N.A. Feasey, C.L. Msefula, M. Alaerts, D.B. Everett, T.J. Allain, R.S. Heyderman);; University of Liverpool, Liverpool (D.B. Everett, M.A. Gordon);; Institute of Food Research, Colney, Norwich, UK (R.A. Kingsley)

**Keywords:** Keywords: Salmonella, serotype Typhimurium, Salmonella enterica, antimicrobial resistance, HIV, Africa, ESBL, fluoroquine resistance, bacteria, Malawi

**To the Editor:** *Salmonella enterica* serotype Typhimurium is one of the most common causes of bloodstream infection in sub-Saharan Africa (*1*). Among adults, the principal risk factor for invasive nontyphoidal *Salmonella* (iNTS) disease is advanced HIV infection; up to 44% of HIV-infected patients experience bacteremic recurrence through recrudescence of the original infection (*2*,*3*). Epidemics of iNTS disease in sub-Saharan Africa have been associated with a novel genotype of *S. enterica* ser. Typhimurium of multilocus sequence type (ST) 313 that is rarely seen outside the region and is associated with multidrug resistance (MDR) to chloramphenicol, cotrimoxazole, and ampicillin (*4*,*5*). As a consequence, ceftriaxone has become a key agent in the empirical management of nonfocal sepsis in Malawi (*6*).

In March 2009, a 40-year-old HIV-infected and antiretroviral therapy–naïve woman sought care in Blantyre, Malawi, with an MDR *S.* *enterica* ser. Typhimurium bloodstream infection. She was treated with ceftriaxone (2 g intravenously once daily) and discharged with oral ciprofloxacin (500 mg twice daily) for 10 days. She was readmitted 1 month later with recurrent fever. At this time, she had an MDR *S. enterica* ser. Typhimurium bloodstream infection with additional resistance to ceftriaxone and ciprofloxacin. In the absence of a locally available effective antimicrobial drug, she was treated with ceftriaxone, gentamicin, and high-dose ciprofloxacin but died shortly thereafter.

To help clarify how this extended MDR *S.* *enterica* ser. Typhimurium emerged, we determined the molecular mechanisms underpinning this disturbing pattern of antimicrobial resistance (Technical Appendix). We conducted phenotypic drug susceptibility testing by disk diffusion on *S.* *enterica* ser. Typhimurium strains A54285 (initial presentation) and A54560 (recurrence); both isolates were resistant to ampicillin, chloramphenicol, and cotrimoxazole, but A54560 exhibited additional resistance to ceftriaxone, ciprofloxacin, and tetracycline.

Paired-end sequencing of isolates A54285 (European Nucleotide Archive [ENA] accession number ERS035867) and A54560 (ENA accession no. ERS035866) that were cultured 1 month apart showed no differences between the conserved regions of these genomes (Figure). The similarity of these *S.* *enterica* ser. Typhimurium genomes strongly suggests that this recrudescence occurred after incomplete clearance of the first infection; although re-infection from the same source is unlikely, it cannot be excluded. Comparison of the accessory genomes, however, showed an additional 300 kb DNA in A54560.

**Figure:**
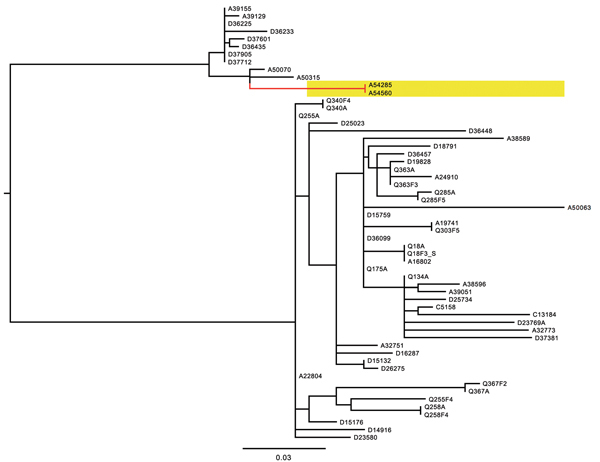
Midpoint-rooted phylogenetic tree of published whole-genome sequence data from D23580-like *Salmonella enterica* serotype Typhimurium sequence type 313s from Malawi based on 204 informative single-nucleotide polymorphisms. A54285 and A54560, highlighted in yellow on a red branch, are indistinguishable. Scale bar indicates nucleotide substitutions per site.

Plasmid extraction and gel electrophoresis of genomic DNA identified a plasmid migrating in the gel to a position approximately equivalent to 120 kb, the size of ST313 virulence plasmid pSLT-BT in both strains, but no 300-kb plasmid was visualized in the ceftriaxone- and ciprofloxacin-resistant strain (A54560, data not shown), possibly because of the difficulty large plasmids have entering standard 1% agarose gels. However, ceftriaxone resistance was mobilized to *Escherichia coli* by conjugation at a frequency 6.5 × 10^−2^ transconjugants per donor at 26°C. This frequency dropped dramatically to ≈1 × 10^−7^ transconjugants per donor when conjugation was performed at 37°C. The presence of an IncHI2 plasmid in the transconjugants was confirmed by PCR for the IncHI2 region (*7*), and drug susceptibility testing confirmed that transconjugant clones acquired resistance to ceftriaxone, ciprofloxacin, and tetracycline. 

These data confirm the presence of an extended-spectrum β-lactamase (ESBL)–producing IncHI2 plasmid in strain A54560 that is capable of conjugative transfer and suggest that the plasmid might have been acquired by residual index strain within the patient by transfer from an unknown donor bacterium. Partial decolonization of the patient’s gastrointestinal tract by ceftriaxone and fluoroquinolone antimicrobial therapy might have rendered it receptive to colonization by ESBL-producing bacteria, which we hypothesize donated the plasmid to the residual index strain.

The transconjugant plasmid DNA was sequenced by using the PacBio RSII platform (Pacific Biosciences, Menlo Park, CA, USA; http://www.pacificbiosciences.com), which assembled as a single contiguous sequence of 309,406 bp, designated pSTm-BTCR (Technical Appendix Figure, ENA accession no. LK056646). We identified 331 predicted coding sequences, including 109 genes required for replication and transfer and 61 genes predicted to be associated with metabolism, membranes, virulence, antimicrobial resistance, and a toxin/antitoxin addiction system. We found an additional 160 predicted, hypothetical genes. Fifteen putative antimicrobial resistance genes were identified, predicted to encode resistance to; tetracycline (*tetA*(C), *tetR*(C)), β-lactams (*bla*_CTX-M15_, *bla*_TEM-1b_, *bla*_OXA-30_), chloramphenicol (*catB3*, *catA1*), aminoglycosides (*strA*, *strB*, *aadA1*, *aacA4*, *aacC3*), ciprofloxacin (*qnrB1*), ulfonamiides (*sul2*), and trimethoprim (*dfrA14*).

In our experience, ESBL and fluoroquinolone-resistant iNTS remain extremely uncommon in Blantyre, Malawi. This is surprising because diverse ESBL genotypes were observed in other members of *Enterobacteriaceae* in Blantyre within a year after ceftriaxone came into common use locally (*8*). That IncHI2 plasmids transfer most efficiently at temperatures <30°C (*9*), a lower temperature than in the human gastrointestinal tract, might explain why the acquisition of ESBL-producing enzymes through IncHI2 plasmids has not been commonly observed within patients with recurrent iNTS disease in this setting. However, rates of transfer might differ when bacteria are growing in the intestine.

The spread of mobile genetic elements that confer antimicrobial resistance among gram-negative organisms is of considerable concern. Wide dissemination of this strain or the IncHI2 (pSTm-BTCR) plasmid among other salmonellae in sub-Saharan Africa would rapidly render iNTS effectively untreatable with currently available antibacterial drugs.

Technical Appendix Detailed methods and plasmid map of pSTm-BTCR.
