# Supplementary material for: Drug Resistance in Salmonella enterica ser. Typhimurium Bloodstream Infection, Malawi
Source: Emerg Infect Dis. 2014 Nov;20(11):1957–9. doi: 10.3201/eid2011.141175 (PMC4214322; doi:10.3201/eid2011.141175)
Supplement: Technical Appendix — Detailed methods and plasmid map of pSTm-BTCR. [file 14-1175-Techapp-s1.pdf]

Article DOI: http://dx.doi.org/10.3201/eid2011.141175

Drug Resistance in *Salmonella enterica* ser. Typhimurium Bloodstream Infection, Malawi

Technical Appendix

Methods

Bacteria

Salmonella Typhimurium A54285 (index multidrug-resistant) and A54560 (recurrence extended multidrug resistant) were isolated from the same patient at initial presentation and at recurrence 1 month later, respectively. Phenotypic drug susceptibility testing was undertaken by the disk diffusion method (Oxoid, Basingstoke, UK). The sequence of *Salmonella* Typhimurium D23580, an invasive isolate of multilocus sequence type ST313, isolated at Queen Elizabeth Central Hospital in 2004 (*1*), was used as a reference for sequence comparison. A rifampicin-resistant mutant of *Escherichia coli* C600 containing no plasmids was used as a recipient for conjugation.

Sequencing and Comparative Genome Analysis

Genomic DNA from bacteria was prepared by Wizard genomic DNA purification kit (Promega, Madison, WI, USA). Short-read Illumina sequencing libraries were prepared, and paired-end sequencing was performed on 2 μg gDNA on a HiSeq2000 platform (Illumina, San Diego, CA, USA) generating 150-bp reads (*2*). The whole-genome sequence of each isolate was assembled by using Velvet (http://www.ebi.ac.uk/~zerbino/velvet, version 1.2.03) and aligned against D23580 and its associated plasmids by using Abacas (version 1.3.1) (*3*). Single-nucleotide polymorphisms (SNPs) were determined with reference to strain D23580 by using SMALT, version 0.5.8 (Wellcome Trust Sanger Institute, Cambridge, UK). Contigs that failed to align with D23580 were considered as potentially unique mobile genetic elements and were further characterized by using BLASTn analysis (http://www.ncbi.nlm.nih.gov/BLAST) to predict whether they were plasmids; if so, in silico PCR was used to determine the plasmid incompatibility group (*4*).

Plasmid Isolation and DNA Extraction

Plasmid DNA was initially extracted by using an alkaline lysis method (Kado and Liu) and plasmids were profiled by visualising on a 1% agarose gel (*5*).

Individual plasmids were extracted following conjugal transfer from *Salmonella* Typhimurium A54560 to rifampicin-resistant *E. coli* K12 recipient strain c600, as described previously (*6*). Donor and recipient strains were mated by mixing 100 µL of overnight cultures on Luria-Bertani agar plates and incubated overnight at 26°C. Cells were cultured on MacConkey agar containing rifampicin (100 µg/mL) to select for *E. coli* and ceftriaxone (4 µg/mL) to select for transconjugants. The number of donor cells was determined by culture on Luria-Bertani agar containing rifampicin alone, and the transfer frequency was determined and the antimicrobial drug resistance phenotype of transconjugants was determined (Oxoid, USA), to ensure all resistances from the donor strain transferred.

Plasmid DNA was extracted from transconjugants by using an alkaline lysis method optimized for large plasmids, as previously described (*7*). PCR-based replicon typing on the transconjugant DNA was used to confirm the incompatibility group suggested by in silico plasmid typing (*4*).

Plasmid Sequencing

The transconjugant plasmid DNA was sequenced by using the PacBio RSII platform (Pacific Biosciences). Gene prediction and annotation was performed using Prokka (http://vicbioinformatics.com).

References

1. Kingsley RA, Msefula CL, Thomson NR, Kariuki S, Holt KE, Gordon MA, et al. Epidemic multiple drug resistant *Salmonella* Typhimurium causing invasive disease in sub-Saharan Africa have a distinct genotype. Genome Res. 2009;19:2279–87 . PubMed http://dx.doi.org/10.1101/gr.091017.109

2. Harris SR, Cartwright EJ, Torok ME, Holden MT, Brown NM, Ogilvy-Stuart AL, et al. Whole-genome sequencing for analysis of an outbreak of meticillin-resistant *Staphylococcus aureus*: a descriptive study. Lancet Infect Dis. 2013;13:130–6 . PubMed http://dx.doi.org/10.1016/S1473-3099(12)70268-2

3. Swain MT, Tsai IJ, Assefa SA, Newbold C, Berriman M, Otto TD. A post-assembly genome-improvement toolkit (PAGIT) to obtain annotated genomes from contigs. Nat Protoc. 2012;7:1260–84 . PubMed http://dx.doi.org/10.1038/nprot.2012.068

4. Carattoli A, Bertini A, Villa L, Falbo V, Hopkins KL, Threlfall EJ. Identification of plasmids by PCR-based replicon typing. J Microbiol Methods. 2005;63:219–28 . PubMed http://dx.doi.org/10.1016/j.mimet.2005.03.018

5. Kado CI, Liu ST. Rapid procedure for detection and isolation of large and small plasmids. J Bacteriol. 1981;145:1365–73. PubMed

6. Cain AK, Liu X, Djordjevic SP, Hall RM. Transposons related to Tn1696 in IncHI2 plasmids in multiply antibiotic resistant *Salmonella enterica* serovar Typhimurium from Australian animals. Microb Drug Resist. 2010;16:197–202 . PubMed http://dx.doi.org/10.1089/mdr.2010.0042

7. Cain AK, Hall RM. Evolution of IncHI2 plasmids via acquisition of transposons carrying antibiotic resistance determinants. J Antimicrob Chemother. 2012;67:1121–7 . PubMed http://dx.doi.org/10.1093/jac/dks004

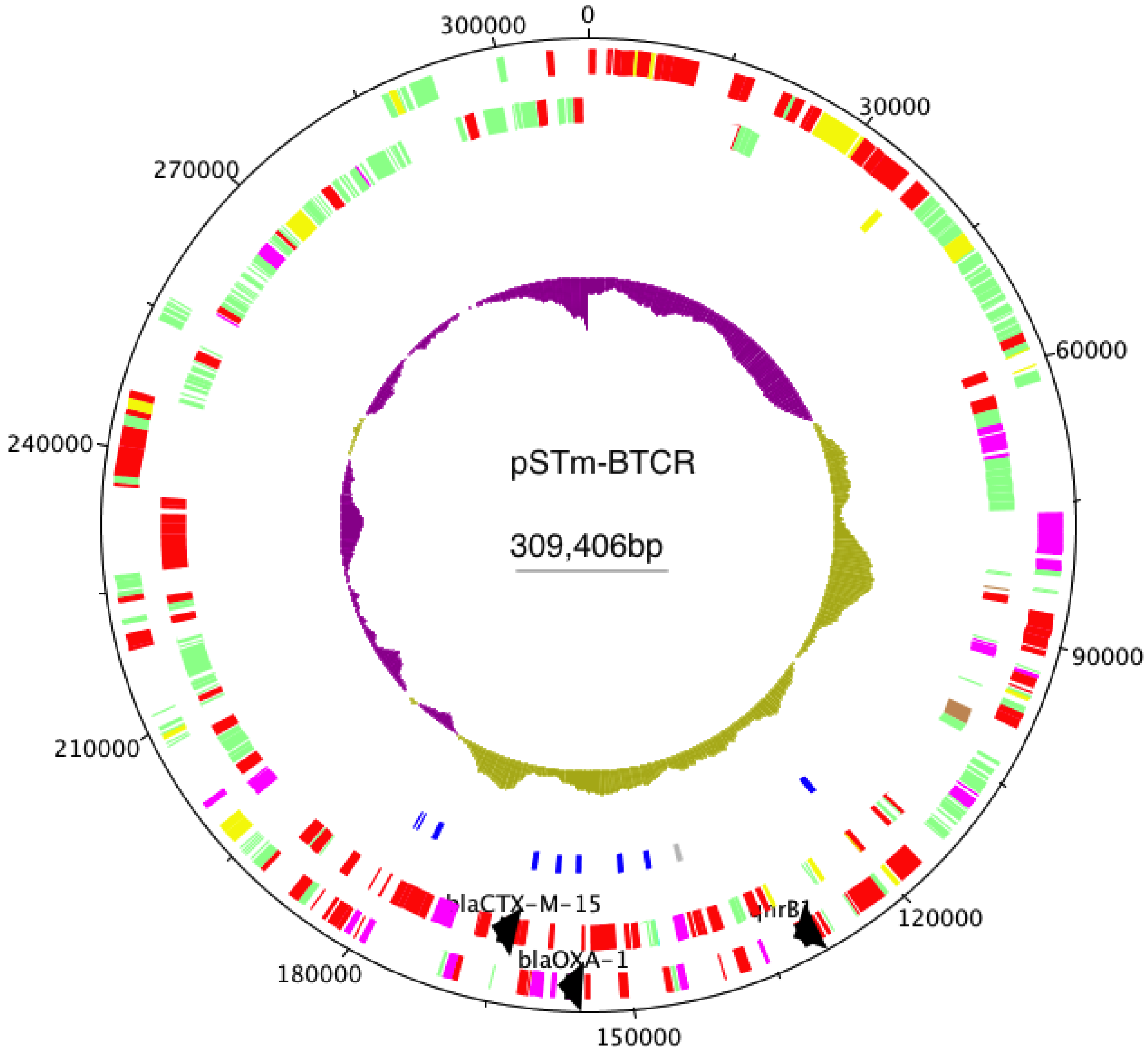

Technical Appendix Figure. Plasmid map of pSTm-BTCR. Circles represent the 309-kbp circular plasmid with *rep1A* close to position 0. The outermost circle shows genes predicted on the forward strand, and the circle inside that shows genes predicted on the reverse strand. Red indicates genes involved in DNA replication; yellow indicates metabolism; purple indicates antimicrobial resistance or virulence; and green marks indicate hypothetical genes. Key resistance genes are annotated. The antimicrobial resistance genes are all located between 128 kbp and 182 kbp. The regions marked in blue in the next track highlights the sites of IS elements. The innermost circle shows the guanine–cytosine plot.
